# ACCESS TO HEALTH CARE FOR LGBT PEOPLE

**DOI:** 10.1093/geroni/igz038.2324

**Published:** 2019-11-08

**Authors:** Katherine Bristowe

**Affiliations:** King’s College London, London, England, United Kingdom

## Abstract

LGBT people have higher risk of life-limiting illnesses and unmet needs when facing advanced illness.The ACCESSCare research programme aims to improve health and social care for LGBT people. ACCESSCare-A explored experiences of 40 UK LGBT people facing serious illness. Discrimination, heteronormative assumptions, and insensitivity influenced whether individuals disclosed relationships to professionals and place-of-care decisions. Professionals must go beyond anti-discrimination to proactive inclusion: 10 evidence-based recommendations were developed to improve care for LGBT people. Our sister study in Zimbabwe explored the healthcare experiences of key populations (LGBTI people, sex workers). Access to healthcare was dependent on conforming to ‘sexual norms’ and care was negatively affected by professionals’ attitudes to key populations. There are two ongoing research projects in the ACCESSCare programme: ACCESSCare-B, a mixed-methods population-based comparative study of LGB and heterosexual bereavement outcomes; and ACCESSCare-C a qualitative study to develop communication guidance for professionals supporting LGBT people facing serious illness.

